# A giant fecaloma causing stercoral colitis secondary to aripiprazole and benztropine: A case report

**DOI:** 10.5339/qmj.2025.58

**Published:** 2025-07-05

**Authors:** Shahem Abbarh, Mhd Kutaiba Albuni, Misbah Irshad, Adnan Humam Hajjar, Bisher Sawaf, Khalid Al-Ejji

**Affiliations:** 1 Department of Internal Medicine, Hamad Medical Corporation, Doha, Qatar; 2 Department of Internal Medicine, Trihealth Good Samaritan Hospital, Cincinnati, OH, USA; 3 Department of Gastroenterology, Hamad Medical Corporation, Doha, Qatar *Email: dr.shahem94@gmail.com

**Keywords:** Antipsychotics, aripiprazole, benztropine, constipation, fecaloma, stercoral colitis

## Abstract

**Background:**

Fecaloma is a mass of hardened feces impacted in the rectum and sigmoid. When the colonic mucosal wall and vasculature are compressed, stercoral colitis, a rare type of inflammatory colitis, may occur. Despite being connected to psychiatric patients and antipsychotic medications in the literature, fecaloma remains a significant, yet often overlooked, cause of morbidity and mortality in this population.

**Case Presentation:**

A 43-year-old patient with schizophrenia being treated with aripiprazole and benztropine lost follow-up and eventually presented to the emergency department with a giant fecaloma and associated stercoral colitis. She was managed with oral and rectal laxatives and supportive therapy. Additionally, aripiprazole was discontinued.

**Discussion:**

Antipsychotics have been associated with constipation, fecaloma, and stercoral colitis due to their anticholinergic properties. Benztropine, an antimuscarinic drug commonly used in psychiatric patients to alleviate extrapyramidal symptoms, may further decrease gastrointestinal mobility. Fecaloma and associated stercoral colitis are often diagnosed via imaging, typically a computed tomography scan of the abdomen. Management depends on the severity and generally ranges from conservative treatment to surgical options.

**Conclusion:**

This case describes a schizophrenic patient undergoing treatment with aripiprazole and benztropine who presented with constipation and was diagnosed with a giant fecaloma complicated by stercoral colitis. The patient was managed successfully with aggressive laxatives and discontinuation of aripiprazole. Early recognition and prompt management of fecaloma are essential to mitigate associated complications. In addition, it is important to recognize risk factors for constipation and regularly review home medications, such as antipsychotics, that may cause constipation as a side effect.

## INTRODUCTION

A fecaloma is a mass of hardened stool and is considered a severe form of stool impaction. Fecaloma can manifest in several ways, including bowel obstruction, urinary bladder or ureter compression,^1^ and toxic megacolon.^2^ It can also cause more serious complications, such as colitis, perforation, peritonitis, or even death.^3,4^ Stercoral colitis is an uncommon entity of inflammatory colitis caused by compression of the colonic mucosal wall and vasculature by fecaloma.^5^

The prevalence of stercoral colitis is not well-established in the literature, owing to its rarity and scarcity of large-scale studies.^6^ There is no specific symptom or sign of stercoral colitis, and its presentation can mimic other acute diagnoses, such as mesenteric ischemia, colon cancer perforation, and appendicitis.^6–8^ Similarly, laboratory investigations are usually only suggestive but not specific. Therefore, when suspecting stercoral colitis, physicians should pursue imaging, typically an erect abdominal X-ray (XR), followed by a computed tomography (CT) scan of the abdomen with contrast, to confirm the diagnosis and guide the management.^5,6^

Factors that lead to fecaloma and stercoral colitis are the same factors that generally contribute to constipation. These factors include decreased mobility, neurologic diseases, and drugs. Many drugs, including antipsychotics, particularly clozapine, have been linked to severe constipation, pseudo-obstruction, and fecaloma.^9^ Benztropine, a commonly used drug alongside antipsychotics to mitigate associated extrapyramidal symptoms (EPS), has also been linked to constipation due to its antimuscarinic characteristics.^10^ Despite being a well-known problem in psychiatry, constipation and its complications, such as fecaloma and stercoral colitis, still happen frequently and cause a real burden.^9^ Therefore, we aim to highlight these preventable complications. Herein, we report a middle-aged woman on antipsychotics for schizophrenia who presented to the emergency department with chronic constipation and abdominal pain.

The case was encountered at Hamad General Hospital, the main public institution providing tertiary healthcare in Qatar. The article was approved by the Institution Review Board at the same facility (MRC-04-25-002). A written informed consent was obtained from the patient to publish all images, clinical data, and other data included in the manuscript. All identifying information has been removed.

## CASE PRESENTATION

We present a 43-year-old woman with a significant past medical history of schizophrenia who presented to our emergency department with chronic constipation, abdominal pain, and distension that have been gradually worsening. She also noticed a decreased appetite and a 15-kg weight loss over the previous 3 months. At the time of schizophrenia diagnosis 3 years previously, the patient was started on monthly paliperidone, then 350 mg intramuscular injection every 3 months. Two weeks after starting 3-month paliperidone, she experienced EPS, evident by sudden involuntary painful contraction of the neck, for which benztropine 2 mg daily was introduced. Add-on aripiprazole 7.5 mg daily was also prescribed as she developed hyperprolactinemia. Six months later, aripiprazole was increased to 15 mg due to persistently rising prolactin levels. After that, the patient was lost to follow-up and stopped taking paliperidone injections, but she continued taking aripiprazole and benztropine until the time of her current presentation. Apart from her psychiatric illness, her past medical history was unremarkable. She denied the use of other home medications, including over-the-counter medicines, herbals, or alcohol. Initial evaluation showed a cachectic weak lady. Vital signs were normal except for a heart rate of 110 beats/minute. Her weight was 45 kg, with a body mass index of 17. Her abdomen was markedly distended and firm on palpation, without rebound tenderness or guarding. Digital rectal examination showed hard impacted stool.

**Figure 1:**
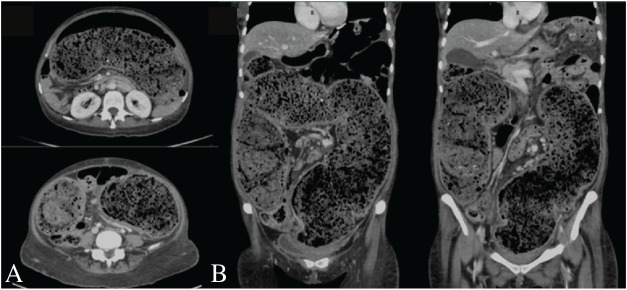
A CT scan of the abdomen and pelvis with IV contrast shown in axial (A) and coronary views (B). Images demonstrating massively dilated large bowel loops due to severe fecal loading occupying the entire abdomen and compressing other abdominal structures. Wall thickening and peri-colonic soft tissue stranding noticed, consistent with stercoral colitis.

Laboratory investigations were significant for iron-deficiency anemia with a hemoglobin level of 8 g/dL. Other investigations revealed urea of 3.6 mml/L, creatinine of 41 μmol/L, sodium of 132 mmol/L, potassium of 3.7 mmol/L, and albumin level at 17 g/L. Liver function tests were normal, including alkaline phosphatase, bilirubin, and liver transaminases. Prothrombin time was 13 seconds, with an international normalized ratio of 1.1. Thyroid-stimulating hormone was 3.6 mU/L. Other laboratory results showed a white blood cell count of 11 × 10^3^/μL, C-reactive protein of 42 mg/L, lactate level of 0.9 mmol/L, and a slightly elevated stool calprotectin at 78 μg/g. Abdominal XR showed impacted stool and air-fluid levels. A CT scan of the abdomen with intravenous contrast revealed massive colonic dilatation with severe fecal loading and bowel wall thickening, suggestive of stercoral colitis (Figure 1).

**Figure 2:**
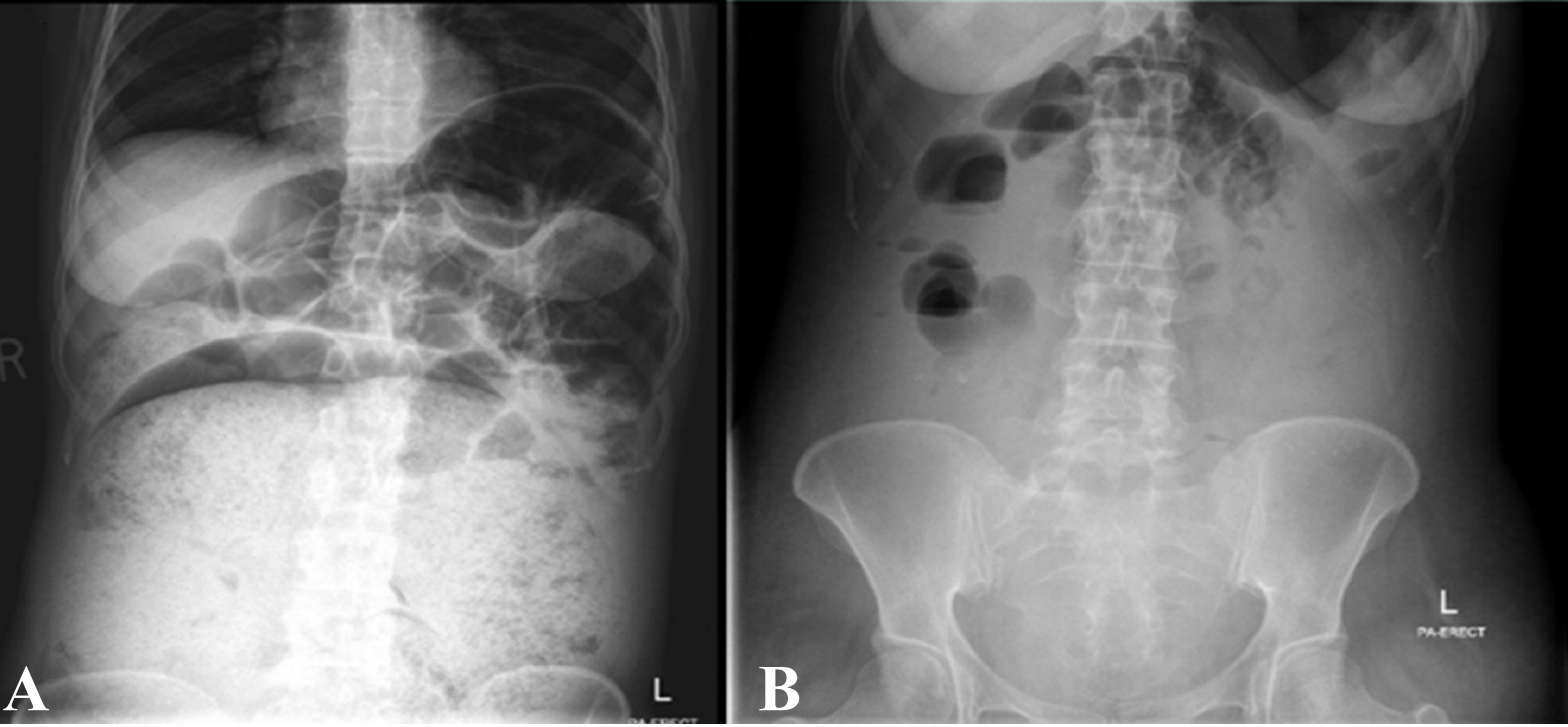
Abdominal XR while standing showing dilated bowel loops filled with fecal material pushing the small bowels and the stomach upwards with air-fluid level (A). After treatment with laxatives, significant improvement of fecal loading and resolution of mass effect on small bowels and stomach noted (B).

The diagnosis of giant fecaloma secondary to aripiprazole and benztropine was made. Gastroenterology and general surgery teams were involved, and they advised conservative management. She was managed with oral laxatives, including senna and polyethylene glycol, rectal enema, intravenous hydration, and nutritional support. Both aripiprazole and benztropine were held. She started to pass bowel motion around two times daily, and within 1 week of treatment, her abdominal distention improved. A follow-up abdominal XR showed an improvement in the previously observed air-fluid levels and reduced fecal impaction (Figure 2). After 2 weeks of conservative and supportive management, she was discharged without complications. The patient was discharged on three laxatives as needed and paliperidone injections. Benztropine was resumed because of the previous history of significant EPS, but aripiprazole was kept on hold. A colonoscopy, done 2 weeks after discharge to evaluate for colonic masses or malignancy, was unremarkable. Multiple colonic biopsies showed few lymphocytes and macrophages in the colonic mucosa, with no other pertinent findings. Six months after discharge, the patient was doing well overall, without psychotic symptoms or significant constipation.

## DISCUSSION

Decreased intestinal mobility is a common but poorly researched side effect of prolonged antipsychotic medication use. Antipsychotics, both typical and atypical, possess weak anticholinergic properties that can lead to constipation, fecaloma, and stercoral colitis.^9^ A well-established literature has connected clozapine to constipation and its complications [11]. Olanzapine, quetiapine, and haloperidol have also been implicated in severe constipation.^3,12^ Aripiprazole and ziprasidone, on the other hand, appear to have the lowest constipation rate among second-generation antipsychotics.^9,12^ This side effect, however, can be exaggerated when antipsychotics are combined with benztropine.

Benztropine is an antimuscarinic drug commonly used in psychiatric patients to alleviate EPS arising from the strong D2 receptor antagonism caused by antipsychotic medications. Due to its anticholinergic effect, benztropine may further decrease gastrointestinal mobility and promote constipation complications. Sheikh RA et al. described a 68-year-old woman who developed acute intestinal pseudo-obstruction following a combination of haloperidol and benztropine.^13^ Similarly, the combination of olanzapine and benztropine has been implicated in constipation and secondary ischemic colitis in a young patient.^14^ Our patient was on a combination of aripiprazole and benztropine. In our case, aripiprazole was used as an adjunctive medicine in the management of antipsychotic-induced hyperprolactinemia,^15^ while benztropine was added to manage EPS, manifested as torticollis. It is difficult to determine which one of them caused the patient’s fecaloma and stercoral colitis. In the literature, we could not find a fecaloma or stercoral colitis case caused by aripiprazole alone or benztropine alone. As both medications may have contributed to severe constipation secondary to their anticholinergic effects, we are proposing that the combination of aripiprazole and benztropine induced the patient’s fecaloma and stercoral colitis.

A fecaloma is a mass of inspissated stool impacted in the colon. It is usually lodged in the sigmoid colon and rectum as the stool hardens and the colon narrows. Fecaloma may present in various ways, including constipation, overflow diarrhea, abdominal distension, abdominal pain (due to colonic wall stretch), and urinary retention/urgency (due to compression of the urinary bladder). In typical cases, the patient would be elderly, with numerous factors contributing to chronic constipation.^16^ These factors include medication-related, such as opioids and antimuscarinics, and inactive state. Nonetheless, symptoms and physical examination of fecaloma are non-specific and may mimic other colonic pathologies, such as diverticulitis, mesenteric ischemia, and malignancy, posing a diagnostic challenge.^16^

Furthermore, fecaloma can be associated with significant morbidity and mortality. Fecaloma impaction can cause colonic injury related to focal pressure necrosis and increased intraluminal pressure, limiting mucosal blood flow. This can result in an uncommon form of inflammatory colitis called stercoral colitis. In a recent large retrospective review of 269 cases of stercoral colitis, nine patients (3.3%) died from a cause related to stercoral colitis within 3 months of diagnosis.^16^ When perforated, fecaloma can be highly deadly. In a study done in France where eight patients were operated on for peritonitis secondary to perforated fecaloma, three patients died in the early postoperative course.^17^ Similarly, in a review study of 64 perforated fecaloma cases, 18 patients (28.1%) died after surgery.^18^ The most commonly reported perforation site is the sigmoid colon.^19^ Death due to fecaloma without perforation has also been reported in the literature.^3,4^ A delay in diagnosing and, thus, managing fecaloma can contribute even to higher morbidity and mortality.

The delay in recognizing fecaloma/stercoral colitis might be owed to the non-specific symptoms and physical examination, along with its rarity. Blood investigations may reveal high inflammatory markers, elevated lactic acid, and metabolic acidosis. However, these lab findings are also unreliable in diagnosing fecaloma/stercoral colitis. Therefore, abdominal imaging is paramount for diagnosing such a condition.^19^ When the clinical picture is highly suspicious of perforation, patients should have an upright chest XR to evaluate for free air under the diaphragm. CT abdomen and pelvis with contrast is the most sensitive and specific imaging to diagnose fecaloma/stercoral colitis. CT Findings that significantly correlate to fatal stercoral colitis include dense mucosa, perfusion defects, ascites, and abnormal gas.^19^

In general, treatment of stercoral colitis depends mainly on the severity of the disease. Conservative management with laxatives, enemas, and manual evacuation is considered adequate in patients who present early without evidence of perforation or severe disease. Endoscopic disimpaction is also reported when conservative measures fail to relieve stool impaction.^20^ Different techniques have also been used, including endoscopic fragmentation with Coca-Cola injection^21^ and jumbo forceps.^22^ The use of Coca-Cola injection to help dissolve and soften the fecaloma was based on its observed beneficial role in managing gastric phytobezoars.^23^ Operative management is reserved for patients with signs of peritonitis secondary to perforation. In cases attributed to antipsychotic medications and/or benztropine use, such as our patient, there are no consensus guidelines regarding discontinuation or dose reduction of implicated medications. Evidence is limited to case reports and is a case-by-case decision based on the patient’s psychiatric background and constipation severity. Our patient was managed conservatively with oral laxatives and enemas. Aripiprazole and benztropine were discontinued on admission. Benztropine was resumed upon discharge after a discussion with the patient, targeting the previous significant EPS.

Prevention of fecaloma would be the best option in light of its morbidity and difficulty to manage in psychiatric patients. First, before prescribing antipsychotics or anticholinergic medications, a thorough medical history regarding risk factors of constipation, including home medications, should be obtained. In addition, patients should be counseled about constipation and advised of lifestyle measures that alleviate constipation, such as adequate fluid intake, a high-fiber diet, and mobility.^24^ Prophylactic laxatives, as needed, should also be prescribed to the patients.

## CONCLUSION

Despite its rarity, fecaloma and associated stercoral colitis may lead to significant morbidity and mortality in psychiatric patients. It should be diagnosed early and managed rapidly to avoid further complications. The presented case was managed successfully with laxatives, supportive therapy, and discontinuing aripiprazole. Other management options may include endoscopic disimpaction and operative management. Finally, reviewing the patient’s home medications and assessing possible side effects is essential in such a condition.

## Conflicts of interest

The authors report no conflict of interest.

## Ethical approval

The article was approved by the Institution Review Board at Hamad Medical Corporation.

## Consent

Informed consent was obtained from the patient for publication of this case. All identifying information has been removed.
